# Notes and News

**Published:** 1894-06-16

**Authors:** 


### Notes and News.

Collected and Communicated.

The Worthing Town Council are going to publish a full illustrated report made by Dr. Kelly on the recent outbreak of typhoid in the town.

On account chiefly of the great inconvenience experienced by the Brazilian quarantine regulations, steps are being taken to see whether regulations adopted by the Dresden Sanitary Convention cannot be adhered to by the Governments of South America. The principal European countries now enforce the regulations formulated at Dresden.

We have received many inquiries as to whether reproductions of the beautiful living pictures shown at the Hospital Sunday conversazione are to be obtained. We wish our readers, and those to whom the subject is of interest, to know that we shall be glad to arrange for the collection, or a selection from it, to be reproduced provided we receive a sufficient number of written applications stating that any or all are desired, addressed to the Editor, 428, Strand, W.C.

The interest of the dust in our streets does not end in the removing dust cart. Dust and street clearings are regarded as matters of importance in this age of the glorification of sanitation. The contagion has spread far and wide, and now we see that Japan will not be behind in the race, for the Japanese Ambassador paid a visit last week to the City dust-yard at Lambeth, to inspect the method pursued by the Commissioners of Sewers for getting rid of the dust and rubbish of our great City.

An amusing notification was made on the programme of the opening ceremony of the Poplar Hospital for Accidents on Monday. This was that "visitors should leave their money or valuables with the chairman or treasurer." Now all things are said to be fair in love and war, and so it would have been well to ask whether "love" and "charity" were to be regarded as synonymous terms on the occasion, that some guarantee for the return of the "money and valuables" might be given, for the wording of the notice seemed open to the interpretation that it was a bold ruse to acquire funds for the hospital

As we go to press, the Hospital Sunday Fund collection for the present year has reached the sum of £9,000, this amount including the magnificent contribution of Canon Fleming's congregation, amounting to upwards of £1,200.

The New York Health Department has decided to pension physicians, nurses, clerks, and others employed by them after twenty years' service, and also to provide pensions for the families of those dying in the discharge of duty.

The forthcoming election to the Council of the Royal College of Surgeons will be fraught with much interest, as competition will be keen, there being only one actual vacancy. Mr. Norton, senior surgeon at St. Mary's Hospital, and Mr. Hardie, from Manchester, are both announced as candidates.

The Burton Infirmary, through Mr. W. H. Worthington, has just benefited to the extent of £10,000. The donor, who is principal member of the well-known brewing firm, imposes the condition that the money shall be invested in debentures or preference stock of some first-rate railway company, the dividends being used for ever towards the maintenance of the institution.

Three eminent professors were nominated to fill the post vacant at Vienna through Dr. Billroth's death. These were Professor Czerny, of Heidelberg; Professor Gussenbauer, of Prague; and Professor Mikulicz, of Breslau. Professor Czerny, however, has retired from the candidature, and it is expected that Professor Mikulicz will be elected. Dr. Escherich is to succeed Dr. Huebner as Professor of Children's Diseases at Leipsic.

A British Committee, of which Sir Douglas Galton, K.C.B., F.R.S., is the chairman, and Professor W. H. Corfield, M.A., M.D. (Oxon), is the treasurer, has been formed to further the interests in this country of the Eighth International Congress of Hygiene and Demography, which is to be held in Budapest from the 1st to the 8th of September this year. Any information may be obtained about the Congress from the Hon. Secretary, Dr. Paul F. Moline, 42, Walton Street, Chelsea, S.W.

We regret to have to report the death of Dr. Robert Jolly, M.D., F.R.C.S.Eng., which occurred at Birmingham on the 9th inst. Dr. Jolly was formerly demonstrator of anatomy at the Royal College of Surgeons at Edinburgh; subsequently for five years house-surgeon at the Queen's Hospital, Birmingham; and then first assistant surgeon and afterwards full surgeon at the General Hospital, Birmingham. At the time of his death Dr. Jolly was senior surgeon of the General Hospital, Birmingham, chairman of its Medical Committee, and consulting surgeon to the General Dispensary. Dr. Jolly was famous in his day as a teacher of anatomy, He had a deservedly high reputation as a surgeon, and it is to be regretted that the claims of a large private practice prevented him from devoting himself entirely to this branch of the profession. Dr. Jolly rose to a foremost place in the Midlands by sheer industry and hard work. He never took any part in the public work of Birmingham, but devoted himself entirely to his patients. For many years Dr. Jolly was surgeon to the Birmingham Police Force, with the members of which he enjoyed a deserved popularity. His death will be regretted by a large circle of patients and many sorrowing friends.

